# Dynamic cyclic fatigue resistance of Reciproc® Blue, One Reci®, R-Motion®, and two Replica-Like endodontic files after autoclave sterilisation and/or immersion in sodium hypochlorite: A comparative *in vitro* study

**DOI:** 10.4317/jced.62529

**Published:** 2025-05-01

**Authors:** Néstor Ríos-Osorio, Sandra Briñez-Rodríguez, Rafael Fernández-Grisales, Juan Triana-Correa, Anthony Pushaina-Velásquez, Hernán García-Restrepo, David Betancur-Calle, Camilo Meza-Meza, Adriana Tapia-Sierra, Carolina Berruecos, José Sierra-Sánchez

**Affiliations:** 1 PhD Program, Biomedical Sciences, Universidad El Bosque, Bogotá-Colombia; 2 Department of Endodontics, Institución Universitaria Colegios de Colombia UNICOC, Bogotá, Colombia; 3 Postgraduate Endodontics Department, School of Dentistry, CES University, Medellin, Colombia

## Abstract

**Background:**

To compare the cyclic fatigue resistance (CFR) of Reciproc® Blue (RB), One Reci® (OR), R-Motion® (RM), Roll Wave Gold (RW) and RCS Blue T (RCS B-T) instruments.

**Material and Methods:**

525 files (105 RB (25/0.08), 105 OR (25/0.06), 105 RM (25/0.06), 105 Wave Roll (25/0.07) and 105 RCS Blue T (25/0.06) were assigned into 7 groups (n =15) for each brand. Group 0: Instruments were not exposed to NaOCl or sterilization. Groups 1 and 4: instruments were exposed to 5% NaOCl 1 and 3 times, respectively. Groups 2 and 5: instruments were sterilized 1 and 3 times, respectively. Groups 3 and 6: Instruments were exposed to both, NaOCl and sterilization 1 and 3 times, respectively. Subsequently, files underwent a dynamic CFR test. The chemical composition of the files’ surfaces from Group 0 was determined using energy-dispersive X-ray spectroscopy (EDS). Cyclic fatigue resistance time was statistically analysed using 1-way ANOVA and the Kruskal-Wallis test. Post hoc multiple range analysis applying Tukey’s test and the Games-Howell test was conducted to assess significant differences.

**Results:**

Greater CFR values were observed in the RB instruments, followed by the RM, OR, RW and RCS B-T files, respectively (*p*<0.05). No significant differences were observed among RM, OR and RW files (*p*>0.05). The lowest CFR values were observed in the RCS B-T files(*p*<0.05). The CFR of RB, RM, and OR decreased when the instruments were autoclaved and immersed in 5% NaOCl (*p*>0.05) (3 times) compared with the control group.

**Conclusions:**

The RB displayed the best CFR values. The RB, RM and OR instruments were more vulnerable to the repeated cycles of NaOCl immersion combined with autoclave sterilisation. The high density of microstructural defects on the surface of RW and RCS B-T instruments, caused by a lack of quality control, makes their biomechanical behaviour unpredictable.

** Key words:**Dynamic cyclic fatigue, nickel-titanium, autoclave sterilisation, sodium hypochlorite, Replica-Like endodontic files.

## Introduction

Nickel-titanium (NiTi) rotary file fracture occurs due to two mechanical deficiencies: torsional fracture or flexural cycle fatigue (1). Several strategies, including file design, surface modifications, electropolishing treatments, and thermal treatments, have been put forth to enhance NiTi files’ flexibility and cyclic fatigue resistance (CFR) (1). Additionally, the reciprocating alternate motion kinematic has improved the overall CFR compared to continuous rotating instruments. The reciprocating kinematic involves a counter-clockwise motion (CCW) to perform the cutting action and a clockwise (CW) movement for releasing the instrument from the dentin walls. This results in lower stress values on the file, thus lowering the risk of plastic deformation and improving CFR (1,2).

Reciproc® Blue (VDW, Munich, Germany), One Reci® (MICRO-MEGA, Besancon, France), and R-Motion® (FKG Dentaire SA, La Chaux-de-Fonds, Switzerland) are some of the best examples of original instruments with reciprocating kinematics. All three systems are built with Control Memory (CM) wire technology. CM-wires are heat-treated NiTi alloys (3). Heat treatments have enabled the development of endodontic files with significant martensitic phase stability, which display greater elasticity and may undergo greater deformation with relatively low stresses than the austenitic phase (1,3).

Reciproc® Blue (RB) files are manufactured by modifying the molecular structure of the NiTi alloy by thermal treatment (martensitic blue, heating 550°C, cooling 120°C), which results in increased flexibility and high CFR. RB features an S-shaped cross-section, two cutting edges, and a non-cutting tip. The RB 0.25/8 displays a regressive taper beginning 3 mm from the tip (variable 8% first 3mm; 4%:3-6mm, and 5%:6-16mm) (1). One Reci® (OR) is a thermally treated (martensitic cobalt, heating 700°C, cooling 120°C) CM-wire system with an electropolished finish. The file’s cross-section is varied and off-centred, with a triple helix tip that gradually evolves into an S-shape towards the shank. The OR 0.25/6 displays a triangular variable triple helix up to 4mm, a 4-6mm transition zone, an S-shape cross-section from 6-16mm, and a constant 6% taper (4). The R-Motion® (RM) system features a non-cutting tip, alternating cutting edges, and a triangle-based symmetrical cross-section design. RM files receive heat treatment (proprietary thermal treatment that favours a phase transition ranging from 32 °C to 35 °C (between martensite and austenite)). RM files undergo an electrochemically polished finish. The RM 0.25/6 displays a constant cross-section and taper (5).

The manufacture of original endodontic systems undergoes rigorous research, development, and production testing before commercialization to ensure controlled quality standards (6). Nonetheless, different companies have begun to manufacture and market instruments that resemble well-known brands’ systems without releasing accurate reports on production and quality control (6,7). These so-called replica-like instruments are sold at comparatively lower prices and share many features with the originals, including nomenclature, colour identification, number, and sequence (9,7). Some of the most recent examples of endodontic replica-like instruments are Roll Wave Gold (RW) (Shenzhen Denco Medical, Shenzhen, China) and RCS Blue T (RCS B-T) (RAMO Medical, Suzhou, China) (6,8).

Endodontic files’ CFR can be impacted by sterilisation cycles and NaOCl solutions. NaOCl corrosion can harm both the physical and mechanical properties of NiTi files by selectively removing nickel from the surface, causing micro-pitting and stress concentrations that lead to or exacerbate crack formation (1). On the contrary, it has been suggested that sterilisation temperatures may impact crystallographic phases in NiTi alloys, leading to enhanced cutting efficiency and CFR (1,9). Still, cyclic fatigue from multiple autoclave sterilisation cycles can lead NiTi files to fracture (10).

Pedulla *et al*. (11), designed and validated a static CFR methodology for heat-treated NiTi instruments following NaOCL immersion and/or sterilisation (11). Performing the cyclic fatigue test under the same experimental design and parameters allows for regulating the interference of multiple variables, hence strengthening its internal validity (6). Recently (2024) our group adapted (to a dynamic test) and applied this protocol in a study comparing the dynamic CFR of RB and WaveOne® Gold (Dentsply Sirona Endodontics, Baillagues, Switzerland) (1). The findings of that study revealed that RB files outperformed WaveOne Gold in terms of CFR. The RB files were more susceptible to cycles of NaOCl immersion or autoclave sterilization. The combined autoclaving and NaOCl immersions had the greatest impact on the mechanical properties of both systems (1).

Given the aforementioned, the primary goal of this study was to compare the CFR of RB, OR, RM and Roll Wave Gold (RW) and RCS Blue T (RCS B-T), following steam sterilization cycles and/or immersion in 5% sodium hypochlorite, adhering to the former Pedulla *et al*. (11) methodological design and protocols, though under dynamic CFR conFiguration.

## Material and Methods

**Figure 1:**
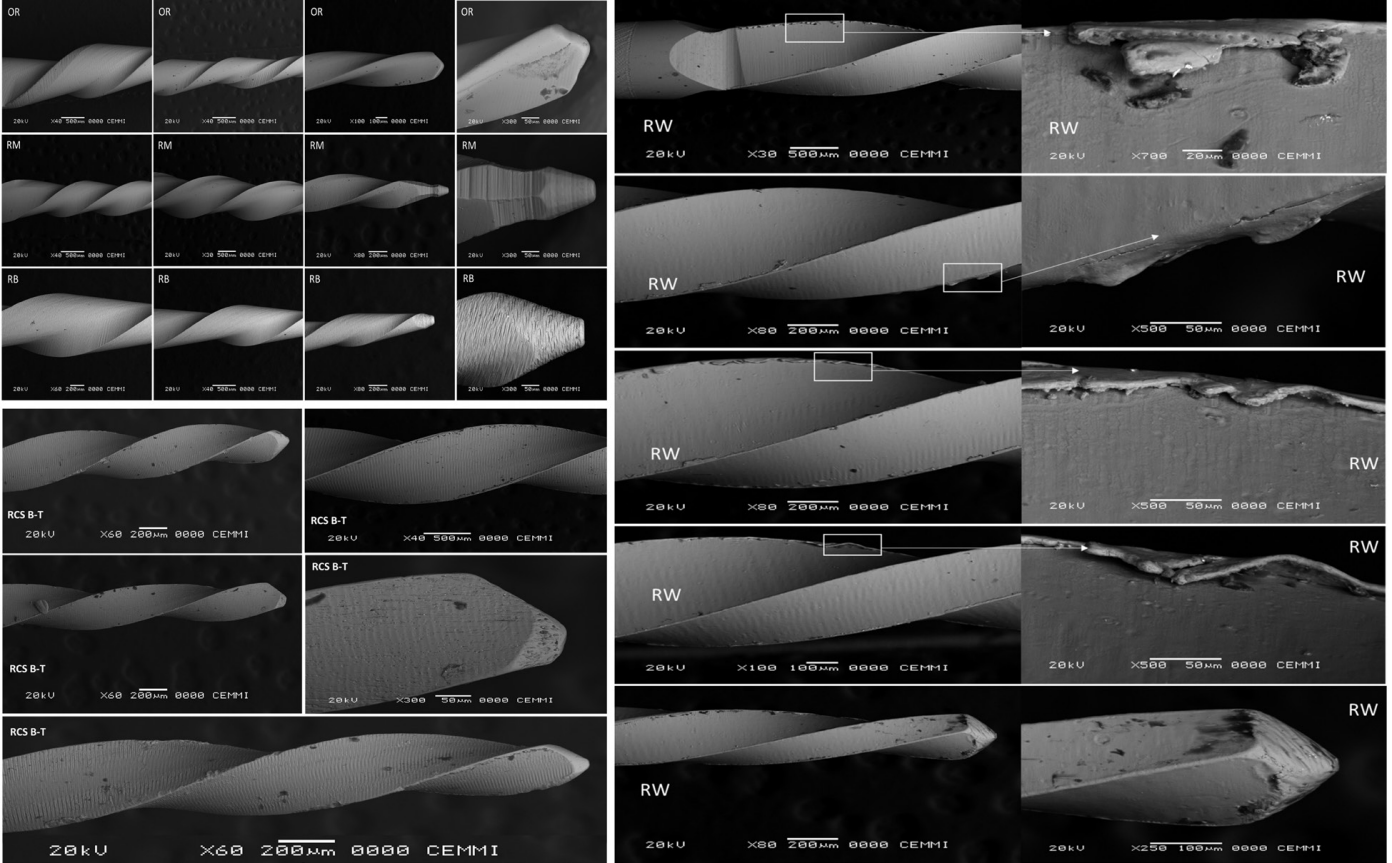
Scanning electron microscope evaluation to verify any signs of visible deformations or microstructural defects of the instruments. No visible defects were observed in any of the RB, OR and RM files. However, high-density microstructural defects, such as machining marks, cracks, pits, pores, scraping and metal rollover, were observed in most of the RW and RCS B-T files. Reciproc Blue (RB), One Reci (OR), R-Motion (RM), RCS Blue T (RCS B-T) and Roll Wave Gold (RW).

The sample size was pre-estimated using the G*Power 3.1.9.2 program (Heinrich-Heine-Universität Düsseldorf in Düsseldorf, Germany) (1,11). Five hundred twenty-five (n=525) endodontic files, 105 RB “R25” (25/0.08), 105 OR (25/0.06), 105 RM (25/0.06), 105 Wave Roll (25/0.07) and 105 RCS Blue T (25/0.06) were tested in this study. All instruments were previously inspected under a stereomicroscope (Leica Zoom 2000, Stereo Microscope, Wetzlar, Germany) and a scanning electron microscope (SEM) at different magnifications (40x to 700x) (JEOL JSM 6460 LV) for any signs of microstructural deformation. There were no visible defects in any of the RB, OR and RM files. However, most of the RW and RCS B-T files presented some kind of structural defect or deformation, thus, it was not possible to replace all of them before the CFR test (Fig. 1). Therefore, all the files under examination underwent the dynamic cyclic fatigue test.

-Study design

The 525 files were distributed randomly to seven groups (11). Group 0 (control group): New instruments that were neither autoclaved nor immersed in NaOCl before the CFR test. Groups 1 and 4: Instruments were dynamically immersed (activated in a VDW Silver Reciproc motor; Munich, Germany (set to RECIPROC ALL)) once and three times, respectively, 16mm deep in 5% NaOCl (ENZOHIP - 5, Prodont Scientific, Bogota, Colombia) at 37˚C for 3 minutes (just the 16-mm active area of the instruments was submerged to prevent galvanic corrosion). After NaOCl immersion, files were flushed with bidistilled water, dried, numbered, and individually stored until the CFR test. In group 4, there was a 30-minute interval between each NaOCL immersion, and instruments were not flushed with bidistilled water between immersions but were washed following the final immersion. Groups 2 and 5: Instruments were autoclaved once and three times, respectively. Instruments were packed separately in sterilising bags. Each sterilisation cycle (Classic autoclave; Star clave, Bogota, Colombia) last 17 minutes / 134˚C. Instruments treated with three steam sterilisation cycles were given time to cool to room temperature and then repackaged following each cycle. Groups 3 and 6: In group 3, instruments were exposed to NaOCl once and sterilised once (as described above). In Group 6, both protocols (NaOCl immersion and sterilisation) were performed thrice. In both groups, sterilisation cycles were performed before 5%NaOCl immersion (11) (Supplement 1) (http://www.medicinaoral.com/medoralfree01/aop/jced_62529_s01.pdf).

-Dynamic CFR test

**Figure 2:**
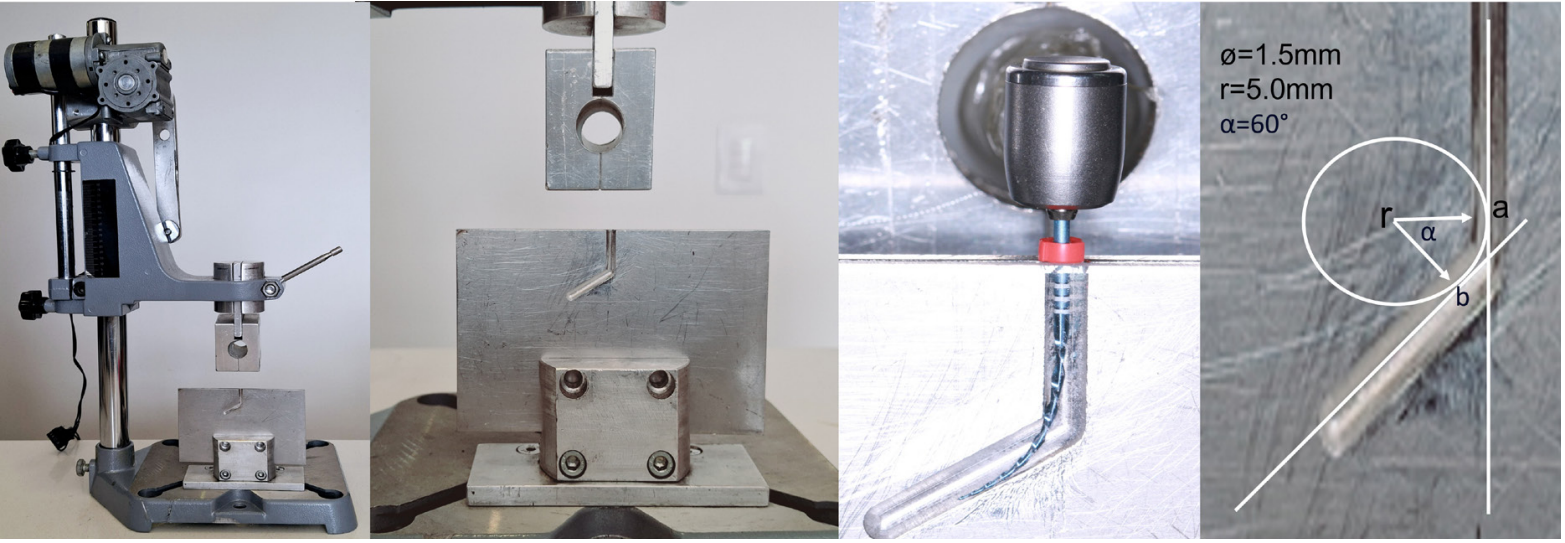
Dynamic cyclic fatigue testing device, stainless-steel device simulating an artificial canal, artificial curved canal with a 60° curvature angle, 5 mm radius and 1.5 mm internal diameter.

**Figure 3:**
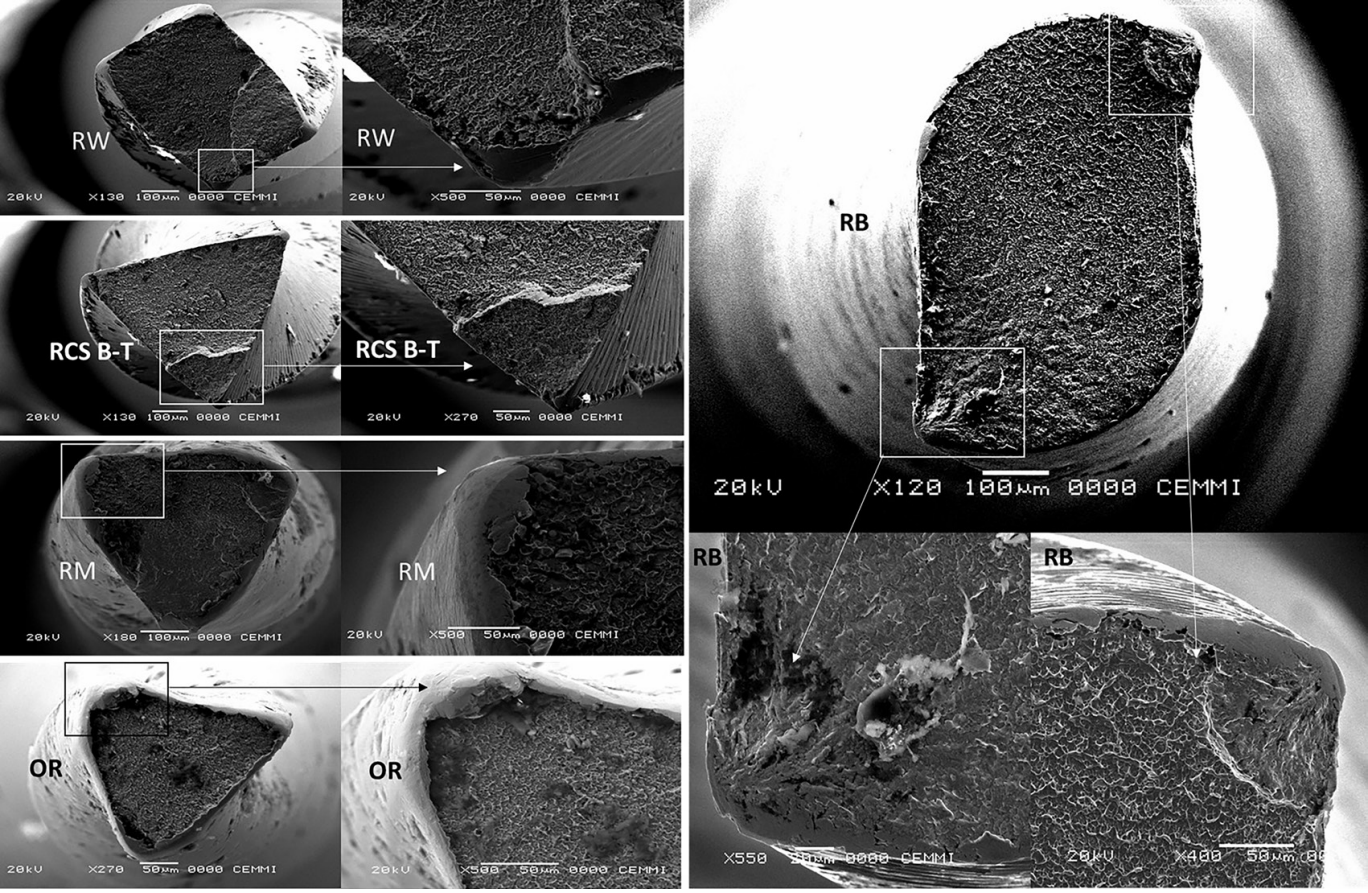
Representative scanning electron microscopic images of fractured specimens (axial views). On the same surface plane, similar fracture patterns (composed of identifiable brittle and plastic areas) are observed. Interestingly, plastic and elastic deformations developed at the fracture’s margins before failure. Reciproc Blue (RB), One Reci (OR), R-Motion (RM), Roll Wave Gold (RW) and RCS Blue T (RCS B-T).

A 300-series stainless-steel canal with a 1.5 mm internal diameter, a 60 ° angle of curvature, and a 5 mm radius of curvature was used for the test (1,11) (Fig. 2). The instruments freely rotate inside the artificial canal under constant pressure, activated in a VDW Silver Reciproc motor (VDW, Munich, Germany), following the manufacturer’s instructions (1,11). Synthetic oil was used to lubricate the artificial canal to reduce instrument friction. The files were operated axially -3 mm/sec- until they fractured. During the CFR test, files inside the canal attained a maximum length of 22 mm (1,11). The fracture time for every tested file was recorded using a timer. Additionally, a video recording of every instrument was set up (1,11). A scanning electron microscope (JEOL JSM 6460 LV) was used to examine the broken instruments (working distance of 9.6 mm, an accelerating voltage of 20 kV). Photomicrographs of the fractured surfaces were captured (Fig. 3) (1,11). A scanning electron microscope (JEOL JSM 6460 LV) with an energy-dispersive X-ray spectroscopy instrument (EDS-Oxford instruments) was used to assess the chemical composition of the files’ surface in the control group before CFR testing. On 1.78kx magnified images, a 100µm2 observation and analysis area was selected for each file (from the middle region). 72 seconds was the effective EDS analysis time.

-Statistical analysis

Data were subjected to the Shapiro-Wilk test following the CRF test to evaluate their normal distribution. The means and standard deviations (SD) of the CFR tests measured in seconds were calculated. The one-way ANOVA test or, in the event that the variable of interest did not have a normal distribution, the Kruskal-Wallis test was employed for the comparison analysis. Post hoc multiple range analysis applying Tukey’s test and the Games-Howell test was conducted to assess significant differences among groups, following the homogeneity of variances analysis. The ANOVA test was used to compare the relative weight percentage (wt.%) of each element across the five file brands. Statistical significance was defined as a *p-value* ˂ 0.05. IBM SPSS® Statistics 29.0 (Armonk, New York: IBM Corp.) software was used to perform the statistical analysis.

## Results

**Figure 4:**
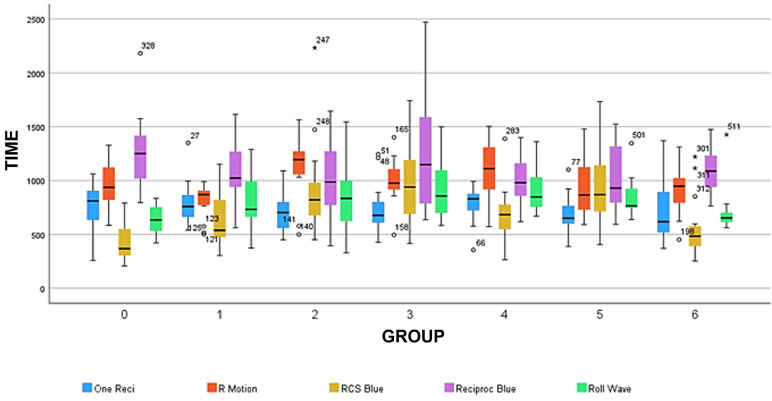
Box Plot analysis of the dynamic cyclic fatigue resistance of the tested instruments after autoclave sterilization and/or immersion in sodium hypochlorite.

 Table 1 shows a descriptive analysis of the mean and standard deviation (SD) of the times recorded in seconds (sec) that correspond to the CFR for each file brand. It offers a comparative analysis that considers the brand of the instruments as an independent variable. The inferential analysis showed statistically significant differences between groups (1-way ANOVA, Tukey or Games Howell, p˂0.05).  Table 2 shows a comparative analysis, considering the treatment as the independent variable. The inferential analysis showed statistically significant differences between the tested instruments (1-way ANOVA, Tukey or Games Howell, p˂0.05). Additionally, the graphic comparison of the dynamic CRF of all the tested instruments under the different experimental conditions (groups) is displayed in Figure 4.

Greater CFR was observed in the RB files followed by the RM, OR, RW and RCS B-T files respectively (p˂0.05) at the control group level. No significant differences were observed among RM, OR and RW files (p˃0.05). The lowest CFR values were observed in the RCS B-T files (p˂0.05) ( Table 2).

Post hoc analysis revealed no differences between the control and all the tested groups of OR, RM and RB files (p˃0.05). Similarly, the RW files show no significant differences between the control and most of the tested groups (1,2,4,5, and 6) (p˃0.05), except group 3, which had a significantly higher CFR than the control group (p˂0.05). Regarding RCS B-T Files, groups 2, 3 and 5 had significantly higher CFR than the control group (Group 0) (*p*<0.05). There were no significant differences between the control group and groups 1,4, and 6 (*p*>0.05) ( Table 1). Although no statistically significant differences were detected, the CFR results for group 6 of RB, RM, and OR show that the combined NaOCl immersion and sterilization (Group 6) had a deleterious effect when compared to the control groups ( Table 1).

The OR (3.43 ± 0.72) and RB (8.46 ± 1.80) were the instruments that fractured in a more apical and coronal position, respectively (*p*<0.05). No significant differences were observed among RM (6.50 ±1.13), RCS B-T (6.84 ± 0.72) and RW (6.25 ± 0.98) (*p*>0.05) ( Table 3).

The EDS analysis revealed that the wt % of the chemical components varied among the evaluated instruments (*p*<0.05). The RB instruments incorporate a higher wt % of C and O than the OR files, a higher wt % of C than the RM and the RCS B-T instruments (*p*<0.05), and a higher wt % of O than the RW instruments (*p*<0.05). The RM files display a higher wt % of O than the rest of the instruments (*p*<0.05).

## Discussion

Although cyclic fatigue is one of the primary causes of instrument failure, several factors such as the angle of the canal curvature, metallurgical characteristics, cross-sectional design, size and taper of the files, pecks/strokes amplitude, and axial and lateral pressure (1,12), rotational speed, angle of instrument insertion, irrigation agents and sterilisation methods may influence instrument fracture. This is especially true since the motor control at least handles the torsional load (12). As a result, all of these parameters were considered when developing and assessing the finding of the current *in vitro* study.

All the systems in this study use a reciprocating kinematic (1,2). We only included martensitic instruments due to the superelastic nature of NiTi instruments in the austenitic phase, which causes them to straighten and press against the outer canal wall, highlighting the significance of alloy characteristics in efficiently controlling curved canals (maintaining the canal’s original anatomy) (1,11). Files were operated in a canal with a 60˚ angle of curvature and a 5 mm radius, allowing for identifying the safest instrument for managing severe curvatures and minimizing the risk of fracture (1,11). Most manufacturers require a vertical amplitude during instrumentation with engine-driven NiTi instruments. Therefore, vertical movements should be incorporated into CFR tests (12,13). The dynamic test conFiguration extends the instrument’s lifetime due to the dispersion of bending strain along the instrument, which avoids a localized load on a single point of the file (12,13). Furthermore, the phase transformation of NiTi alloys dispersed throughout the instrument avoids microcrack development at a particular location of the file (12-14). Consequently, we performed a dynamic CFR test with a 3mm/sec peck amplitude, as specified by most manufacturers of the instruments tested in this study. Hirano *et al*. (15) reported that a 3 mm pecking amplitude improved both the canal-centering ability and CFR of reciprocating instruments while increasing upward (screw-in) and downward forces. Additionally, we standardised the angle of instrument insertion into the canal (straight line), avoiding any lateral movement which could cause torsional loads. A lateral movement of a rotating file may result in a second bending point at the beginning of the canal, confounding the results of dynamic tests (12,13). The methodological design of the present study was validated by Pedulla *et al*. (16), who compared the effects of different access angles and curvature radii on the CFR of NiTi instruments. They concluded that the synergistic effect of a reduced radius of curvature and access angulation on heat-treated instruments lowers their CFR. Accordingly, the results of our study allow us to suggest that RB followed by RM are the most suitable instruments for the management of moderate and severe curvatures; however, the angles of access to the root canals must be kept as straight as possible, as this significantly improves the CFR of the instruments. Our findings are comparable with a recent *in vitro* study by Bürklein *et al*. (17), that assessed the CFR of RB, RM, WaveOne Gold (Dentsply Maillefer, Ballaigues, Switzerland), and Procodile (Komet, Lemgo, Germany) and reported that the CFR of RB 0.25/8 and RM 0.25/6 outperformed the other instruments. No significant differences were found between RB and RM. These authors also reported a superior CFR performance of heat treatments, resulting in blue-coloured instruments. However, unlike our study, it was conducted at body temperature, allowing for a more accurate extrapolation of the results to the clinical setting (17).

We assessed instruments with a comparable size and taper (0.25/6). Instruments of different diameters have varying degrees of curvature in the artificial canals, which might impact the interpretation of the results. However, some of the commercial brands (RB 0.25/8 and RW 0.25/7) presented a different taper than the rest of the instruments, which could suggest a limitation of the present study due to the different increase in the core mass diameter between the instruments. Files with a greater taper display an increased core mass. Instruments with lower core mass experience less stress at the maximum bending points (12). However, results from this study show the greatest CFR values in the RB files (*p*<0.05). Although the RB files had the largest outside diameter, instruments with similar outside diameters at the same section may display different core diameters (12). S-shaped (RB) and triangular cross-sectional designs (RCS B-T) provide lower core mass, which correlates to lower development stress and improved CFR (12). Furthermore, S-shaped cross-sections provide lower core metal mass at the highest stress points (12,18). Additionally, during CFR testing, NiTi instruments exhibit varying deformation reactions based on their cross-sectional design. Instruments with fewer cutting areas, as in the case of RB instruments, face lower stress levels (13). Our findings are consistent with previous research, indicating that S-shaped designs outperform similar-sized instruments in cyclic fatigue tests (12,19-21).

In contrast to the two-stage transformation behaviour (austenite - R-phase - martensite) undergone by gold thermomechanical treatment (OR and RW), which results in finely dispersed Ti3Ni4 precipitates in the austenitic matrix and an austenite finish temperature (Af) higher than 50°C, the blue thermomechanical treatment, seen in RB, RM, and RCS B-T files, has been linked to a single-stage transformation (austenite-martensite) and a lower Af (38.4 ± 0.6°C) which suggests that the alloy goes through reverse transformation via the R phase. Thus, a sTable martensite phase may also help explain the higher CFR displayed by the blue thermomechanical treated files (RB and RM) (1,22). Although RCS B-T files also feature a triangular cross-sectional design (lower core mass) and blue heat treatment, these files exhibited the lowest resistance to the dynamic CFR test observed in this study. Most of the Replica-Like endodontic files (RCS B-T and RW instruments) exhibited microstructural defects such as machining marks, cracks, pits, pores, scraping and metal rollover on the surface during SEM analysis before CFR testing (Fig. 1). The high density of surface imperfections promotes crack nucleation, which leads to fatigue failure by crack propagation, thus impacting CFR and making the biomechanical behaviour of these instruments unpredicTable. To the best of our knowledge, only one previous study has assessed the physicomechanical properties of RW instruments (6). The study’s results are consistent with our findings in that when examined under SEM, RW instruments showed a substantial number of microstructural defects, mainly porosities (6). No previous study assessed the physicomechanical properties of RCS B-T instruments.

EDS analysis showed that the RB instruments incorporate a higher wt % of C and O than the OR files, a higher wt % of C than the RM and the RCS B-T instruments (*p*<0.05), and a higher wt % of O than the RW instruments (*p*<0.05). The RM files display a higher wt % of O than the rest of the instruments (*p*<0.05). Similar findings were previously reported by Rios-Osorio *et al*. (1). These results could offer yet another convincing justification for RB and RM instruments’ higher CFR. Allotropic in nature, titanium can display body-centred cubic (β or martensite) or compact hexagonal (α or austenite) forms. The alloying elements of titanium can be classified as either neutral (betagenic or α-phase stabilizers) or alphagenic (β-phase stabilizers) based on the stabilizing effects of the α and β phases. Phase stabilization refers to a greater or reduced transition temperature β (23,24). Alphagenic elements elevate the transition temperature β. The most important alloying elements among the alphagenic elements are Al, C, and O. Consequently, a more martensitic crystalline structure is produced when these elements are present in large concentrations, as in the case of RB and RM instruments, which increases the flexibility and fracture resistance of alloys, thus leading to a higher CFR as observed in the present *in vitro* study ( Table 4) (1,23,24).

The OR (3.43 ± 0.72) and RB (8.46 ± 1.80) were the instruments that fractured in a more apical and coronal position, respectively. The OR instrument’s apical fractures were regularly noted around 4 apical millimetres when the instrument began to shift from a triangular triple helix cross section with a larger core mass to an S-shaped profile with a lower core mass. Despite keeping a constant taper and variable helix angle, this design alteration most certainly affected the instrument’s fatigue resistance. Notably, the fracture in the OR files occurred below (apically) the artificial canal’s maximal curvature. These findings have clinical implications, as the more apical the fracture of the endodontic instrument (especially in the presence of a root curvature), the complexity and risk of removing and/or overpassing the fractured fragment increases, which can jeopardize the outcome of the endodontic treatment, especially in the presence of apical lesions (25). However, in dynamic testing, fragment length fluctuates based on the position of the centre of curvature (12). Interestingly, SEM examination of fractured fragments regularly revealed regions where deformation took place at the fracture’s margins before failure. These findings demonstrate how plastic and elastic deformations can develop before fractures, enabling clinicians to dispose of instruments before catastrophic failures (Fig. 3).

The high temperatures and pressure generated during sterilisation cycles may cause a change in metallurgical properties as the alloy transitions from the martensite phase to the stiffer austenite phase (1,26). Autoclaving can corrode the surface of the endodontic files, with a cumulative effect on the structure of the rotary NiTi instruments. Furthermore, several autoclave cycles can increase the depth of NiTi instruments’ surface microstructural defects, which increases crack propagation (1,27). NaOCl may remove Ni from NiTi alloys, influencing the physical and mechanical properties of the instruments (1,28). This pitting corrosive behaviour of NaOCl develops owing to the anodic current density profile of NiTi alloys (up to -h300 mV). In the presence of NaOCl, a drop in anodic current density and polarity inversion (cathodic to anodic) are suggestive of various corrosion potentials responsible for the surface modifications (29). The findings of this study show that unlike the RW and RCS B-T instruments, the CFR of the three original endodontic systems (RB, RM, and OR) decreased when the instruments were -autoclaved and immersed in NaOCl- (3 times), showing a synergistic effect between the two treatments. This, together with the plastic and elastic deformation that develops after each use, suggests that the instruments are best suited for maximal use twice. Similar results regarding RB and WaveOne Gold were recently published (1). On the other hand, the results of this study showed that autoclave sterilization (1 and 3 times) significantly increased the CFR of the RCS B-T instruments. It has been suggested that the influence of sterilization cycles on mechanical properties varies according to the type of file (30). Notably, only instruments that have been thermally treated throughout the final manufacturing process can be expected to benefit from sterilization. Because no thermal treatment is undertaken after machining, the instruments’ surfaces are likely to display sTable martensite with a large concentration of dislocations (lattice defects). Sterilization methods produce dislocation annihilation and the shift of stabilized martensite back to austenite. Thus, the observed increase in the CFR of the RCS B-T instruments (which display a high density of microstructural defects) subjected to sterilization may be simply the result of a delay in fatigue crack nucleation and/or propagation (30).

This study’s primary limitation may be that the CFR test ought to be carried out at body temperature (37°C), as heat-treated instruments show noTable stability of the martensitic phase at this temperature (9). Nevertheless, there is still debate because some recent studies did not discover that temperature variations affected CFR values in any way (12,31-33). However, instruments of similar manufacturing, kinematic size and taper were tested under similar and controlled settings, lending validity to the comparison.

## Conclusions

Greater CFR values were observed in the RB instruments, followed by the RM, OR, RW and RCS B-T files, respectively. No significant differences were observed among RM, OR and RW files. The lowest CFR values were observed in the RCS B-T files. RB is the safest instrument for handling moderate to severe canal curvatures, followed by RM instruments. The CFR of RB, RM, and OR decreased when the instruments were autoclaved and immersed in 5% NaOCl (3 times). autoclave sterilization (1 and 3 times) significantly increased the CFR of the RCS B-T instruments. The high density of microstructural defects on the surface of RW and RCS B-T instruments makes their biomechanical behaviour unpredicTable in both *in vitro* and clinical settings. The emergence of replica-like endodontic instruments and the lack of technical information provided by the instrument manufacturer necessitate the evaluation of each biomechanical aspect of these instruments to allow their controlled and conscious use.

## Figures and Tables

**Table 1:** Means and Standard Deviations (SD) of time (expressed in seconds) of the dynamic CFT test for the tested instruments in all groups, considers the brand of the instruments as an independent variable.

		One Reci	R Motion	RCS Blue T	Reciproc Blue	Roll Wave		Total
Group	n	Mean (ED)	Mean (ED)	Mean (ED)	Mean (ED)	Mean (ED)	n	
0	15	769 ± 214^a^	968 ± 230^ab^	436 ± 176^a^	1246 ± 359^a^	630 ± 131^a^	75	810 ± 362^a^
1	15	794 ± 203^a^	807 ± 155^a^	647 ± 249^a^	1098 ± 264^a^	808 ± 254^ab^	75	831 ± 267^ab^
2	15	711 ± 194^a^	1126 ± 273^b^	925 ± 446^b^	1036 ± 357^a^	835 ± 315^ab^	75	927 ± 351^ab^
3	15	735 ± 233^a^	998 ± 208^ab^	979 ± 404^b^	1292 ± 560^a^	933 ± 287^b^	75	987 ± 39^ c^
4	15	788 ± 162^a^	1091 ± 275^b^	686 ± 266^a^	1017 ± 226^a^	910 ± 204^ab^	75	898 ± 269^abc^
5	15	687 ± 185^a^	930 ± 251^ab^	941 ± 362^b^	1036 ± 327^a^	850 ± 177^ab^	75	889 ± 288^abc^
6	15	702 ± 279^a^	916 ± 229^ab^	559 ± 285^ac^	1089 ± 197^a^	703 ± 208^ab^	75	794 ± 30^ ab^
Total	105	741 ± 211	976 ± 249	739 ± 372	1116 ± 350	810 ± 248	525	877 ± 327
p-value		0.716	0.007	<0.001	0.188	0.006		0.002

SD: Standard Deviation. The same letters show differences not statistically significant (*p*>0.05) in comparison with different groups of the same instrument; different letters show differences statistically significant (*p*<0.05) in comparison with the different groups of the same instrument.

**Table 2:** Means and Standard Deviations (SD) of time (expressed in seconds) of the dynamic CFT test for the tested instruments in all groups, considering the treatment (Groups) as the independent variable.

		One Reci	R Motion	RCS Blue T	Reciproc Blue	Roll Wave		Total	p-value
Group	n	Mean (ED)	Mean (ED)	Mean (ED)	Mean (ED)	Mean (ED)	n		
0	15	769 ± 214^a^	968 ± 230^a^	436 ± 176^b^	1246 ± 359^c^	630 ± 131^a^	75	810 ± 362	<0.001
1	15	794 ± 203^ a^	807 ± 155^ a^	647 ± 249 ^a^	1098 ± 264^b^	808 ± 254^a^	75	831 ± 267	<0.001
2	15	711 ± 194^a^	1126 ± 273^b^	925 ± 446 ^ab^	1036 ± 357^ab^	835 ± 315^ ab^	75	927 ± 351	0.009
3	15	735 ± 233^a^	998 ± 208^ ab^	979 ± 404^ab^	1292 ± 560^b^	933 ± 287^ab^	75	987 ± 396	0.003
4	15	788 ± 162^a^	1091 ± 275^b^	686 ± 266^a^	1017 ± 226^b^	910 ± 204^ab^	75	898 ± 269	<0.001
5	15	687 ± 185^a^	930 ± 251^ab^	941 ± 362^ab^	1036 ± 327^b^	850 ± 177^ ab^	75	889 ± 288	0.012
6	15	702 ± 279^a^	916 ± 229^ ab^	559 ± 285^a^	1089 ± 197^b^	703 ± 208^ a^	75	794 ± 301	<0.001
Total	105	741 ± 211^a^	976 ± 249^ b^	739 ± 372^a^	1116 ± 350^ c^	810 ± 248^ a^	525	877 ± 327	<0.001

SD: Standard Deviation. The same letters show differences not statistically significant (*p*>0.05) in comparison with different groups of the same instrument; different letters show differences statistically significant (*p*<0.05) in comparison with the different groups of the same instrument.

**Table 3:** Summary of the statistical and comparative analysis of the measurements where the most apical fracture occurred according to the brand of the files. Means and Standard Deviations (SD) of length (expressed in millimetres).

	One Reci	R Motion	RCS Blue T	Reciproc Blue	Roll Wave	Total	p-value
Apical fragment Mean (ED)	3.43 ± 0.72^ a^	6.50 ±1.13^ b^	6.84 ± 0.72^ b^	8.46 ±1.80^ c^	6.25 ± 0.98^b^	6.30 ± 1.99	< 0.01

SD: Standard Deviation. The same letters show differences not statistically significant (*p*>0.05) in comparison with different instruments; different letters show differences statistically significant (*p*<0.05) in comparison with different instruments.

**Table 4:** Means and standard deviations (SD) of elemental analysis (EDX) of the external surface of the tested instruments.

	One Reci	R Motion	RCS Blue T	Reciproc Blue	Roll Wave	P-value
Weight%	Mean (ED)	Mean (ED)	Mean (ED)	Mean (ED)	Mean (ED)	
C	0.0 ± 0.0^ a^	0.0 ± 0.0^ a^	0.0 ± 0.0^ a^	1.05 ± 0.58^ b^	1.02 ± 0.52^b^	<0.001
Ni	51.33 ± 0.61^a^	48.03 ± 0.55^b^	48.87 ± 0.44^ c^	48.06 ± 0.73^ bd^	48.77 ± 0.55^ bcd^	<0.001
O	6.26 ± 1.36^a^	12.54 ± 1.07^b^	10.64 ± 0.64^ c^	11.17 ± 0.79^c^	9.26 ± 1.08^ d^	<0.001
Si	0.0 ± 0.0^a^	0.0 ± 0.0^ a^	0.0 ± 0.0^a^	0.04 ± 0.12^a^	0.0 ± 0.0^ a^	0.471
Ti	42.42 ± 0.87^a^	39.43 ± 0.66^b^	40.50 ± 0.35^c^	39.63 ± 0.52^b^	40.95 ± 0.68^ c^	<0.001

SD: Standard Deviation. The same letters show differences not statistically significant (*p*>0.05) in comparison with the same chemical element of the different instruments; different letters show differences statistically significant (*p*<0.05) in comparison with the same chemical elements of the different instruments.

## Data Availability

The datasets used and/or analyzed during the current study are available from the corresponding author.

## References

[1] Ríos-Osorio N, Caviedes-Bucheli J, Murcia-Celedón J, Gutiérrez C, Sierra-Collazo D, Alvarado-Caicedo B (2024). Comparison of dynamic cyclic fatigue resistance of Reciproc® Blue and WaveOne® Gold after sterilization and/or immersion in sodium hypochlorite. J Clin Exp Dent.

[2] Ferreira F, Adeodato C, Barbosa I, Aboud L, Scelza P, Zaccaro Scelza M (2017). Movement kinematics and cyclic fatigue of NiTi rotary instruments: a systematic review. Int Endod J.

[3] Tabassum S, Zafar K, Umer F (2019). Nickel-Titanium Rotary File Systems: What's New?. Eur Endod J.

[4] Harorlı H, Koç S, Kuştarcı A (2024). Extrusion of debris during retreatment using various nickel-titanium files in teeth with simulated lateral root perforation. J Oral Sci.

[5] Basturk FB, Özyürek T, Uslu G, Gündoğar M (2022). Mechanical Properties of the New Generation RACE EVO and R-Motion Nickel-Titanium Instruments. Materials (Basel).

[6] Ragozzini G, Abu Hasna A, Dos Reis FAS, de Moura FB, Campos TMB, Bueno CES (2024). Effect of Autoclave Sterilization on the Number of Uses and Resistance to Cyclic Fatigue of WaveOne Gold and Four Replica-Like Endodontic Instruments. Int J Dent.

[7] Martins JNR, Silva EJNL, Marques D, Pereira MR, Ginjeira A, Silva RJC (2020). Mechanical Performance and Metallurgical Features of ProTaper Universal and 6 Replicalike Systems. J Endod.

[8] (2024). Ramo Medical - RCS Blue - Ramo.

[9] Bulem UK, Kececi AD, Guldas HE (2013). Experimental evaluation of cyclic fatigue resistance of four different nickel-titanium instruments after immersion in sodium hypochlorite and/or sterilization. J Appl Oral Sci.

[10] Al-Amidi AH, Al-Gharrawi HA (2023). Effect of autoclave sterilization on the cyclic fatigue resistance of EdgeFile X7, 2Shape, and F-one nickel-titanium endodontic instruments. J Conserv Dent.

[11] Pedullà E, Benites A, La Rosa GM, Plotino G, Grande NM, Rapisarda E (2018). Cyclic Fatigue Resistance of Heat-treated Nickel-titanium Instruments after Immersion in Sodium Hypochlorite and/or Sterilization. J Endod.

[12] Schäfer E, Bürklein S, Donnermeyer D (2022). A critical analysis of research methods and experimental models to study the physical properties of NiTi instruments and their fracture characteristics. Int Endod J.

[13] Hülsmann M, Donnermeyer D, Schäfer E (2019). A critical appraisal of studies on cyclic fatigue resistance of engine-driven endodontic instruments. Int Endod J.

[14] Zupanc J, Vahdat-Pajouh N, Schäfer E (2018). New thermomechanically treated NiTi alloys - a review. Int Endod J.

[15] Hirano K, Kimura S, Maki K, Omori S, Ebihara A, Okiji T (2025). Impact of Varying Amplitudes of Upward and Downward Motion on the Torque/Force Generation, Canal-Centering Ability, and Cyclic Fatigue Resistance of Nickel-Titanium Reciprocating Instrument. Appl. Sci.

[16] Pedullà E, La Rosa GRM, Virgillito C, Rapisarda E, Kim HC, Generali L (2020). Cyclic Fatigue Resistance of Nickel-titanium Rotary Instruments according to the Angle of File Access and Radius of Root Canal. J Endod.

[17] Bürklein S, Maßmann P, Schäfer E, Donnermeyer D (2024). Cyclic Fatigue of Different Reciprocating Endodontic Instruments Using Matching Artificial Root Canals at Body Temperature In Vitro. Materials (Basel).

[18] Kaval ME, Capar ID, Ertas H, Sen BH (2017). Comparative evaluation of cyclic fatigue resistance of four different nickel-titanium rotary files with different cross-sectional designs and alloy properties. Clin Oral Investig.

[19] Di Nardo D, Gambarini G, Seracchiani M, Mazzoni A, Zanza A, Giudice A (2020). Influence of different cross-section on cyclic fatigue resistance of two nickel-titanium rotary instruments with same heat treatment: An in vitro study. Saudi Endodontic Journal.

[20] Sekar V, Kumar R, Nandini S, Ballal S, Velmurugan N (2016). Assessment of the role of cross section on fatigue resistance of rotary files when used in reciprocation. Eur J Dent.

[21] Cheung GS, Zhang EW, Zheng YF (2011). A numerical method for predicting the bending fatigue life of NiTi and stainless steel root canal instruments. Int Endod J.

[22] Hou XM, Yang YJ, Qian J (2020). Phase transformation behaviors and mechanical properties of NiTi endodontic files after gold heat treatment and blue heat treatment. J Oral Sci.

[23] Seracchiani M, Miccoli G, Di Nardo D, Zanza A, Cantore M, Gambarini G (2020). Effect of flexural stress on torsional resistance of NiTi instruments. J. Endod.

[24] Faus-Matoses V, Pérez García R, Faus-Llácer V, Faus-Matoses I, Alonso Ezpeleta Ó, Albaladejo Martínez A (2022). Comparative Study of the SEM Evaluation, EDX Assessment, Morphometric Analysis, and Cyclic Fatigue Resistance of Three Novel Brands of NiTi Alloy Endodontic Files. Int J Environ Res Public Health.

[25] McGuigan MB, Louca C, Duncan HF (2013). The impact of fractured endodontic instruments on treatment outcome. Br Dent J.

[26] Al-Amidi AH, Al-Gharrawi HA (2023). Effect of autoclave sterilization on the cyclic fatigue resistance of EdgeFile X7, 2Shape, and F-one nickel-titanium endodontic instruments. J Conserv Dent.

[27] Valois CR, Silva LP, Azevedo RB (2008). Multiple autoclave cycles affect the surface of rotary nickel-titanium files: an atomic force microscopy study. J Endod.

[28] Peters OA, Roehlike JO, Baumann MA (2007). Effect of immersion in sodium hypochlorite on torque and fatigue resistance of nickel-titanium instruments. J Endod.

[29] Sarkar NK, Redmond W, Schwaninger B, Goldberg AJ (1983). The chloride corrosion behaviour of four orthodontic wires. J Oral Rehabil.

[30] Viana AC, Gonzalez BM, Buono VT, Bahia MG (2006). Influence of sterilization on mechanical properties and fatigue resistance of nickel-titanium rotary endodontic instruments. Int Endod J.

[31] Arias A, Macorra JC, Govindjee S, Peters OA (2018). Correlation between Temperature-dependent Fatigue Resistance and Differential Scanning Calorimetry Analysis for 2 Contemporary Rotary Instruments. J Endod.

[32] Plotino G, Grande NM, Mercadé Bellido M, Testarelli L, Gambarini G (2017). Influence of Temperature on Cyclic Fatigue Resistance of ProTaper Gold and ProTaper Universal Rotary Files. J Endod.

[33] Keleş A, Eymirli A, Uyanık O, Nagas E (2019). Influence of static and dynamic cyclic fatigue tests on the lifespan of four reciprocating systems at different temperatures. Int Endod J.

